# B-type lamins in health and disease^☆^

**DOI:** 10.1016/j.semcdb.2013.12.012

**Published:** 2014-05

**Authors:** C.J. Hutchison

**Affiliations:** School of Biological and Biomedical Sciences, Durham University, Durham DH1 3LE, United Kingdom

**Keywords:** Lamin B1, Cellular senescence, Brain development, Autosomal dominant leukodystrophy

## Abstract

For over two decades, B-type lamins were thought to have roles in fundamental processes including correct assembly of nuclear envelopes, DNA replication, transcription and cell survival. Recent studies have questioned these roles and have instead emphasised the role of these proteins in tissue building and tissue integrity, particularly in tissues devoid of A-type lamins. Other studies have suggested that the expression of B-type lamins in somatic cells influences the rate of entry into states of cellular senescence. In humans duplication of the LMNB1 gene (encoding lamin B1) causes an adult onset neurodegenerative disorder, termed autosomal dominant leukodystrophy, whilst very recently, LMNB1 has been implicated as a susceptibility gene in neural tube defects. This is consistent with studies in mice that reveal a critical role for B-type lamins in neuronal migration and brain development. In this review, I will consider how different model systems have contributed to our understanding of the functions of B-type lamins and which of those functions are critical for human health and disease.

## Highlights

• The role of B-type lamins in cellular organisation and function.
• The role of B-type lamins in chromatin organisation.
• The role of lamin B1 in cellular senescence.
• The role of B-type lamins in organogenesis and tissue building.

## 1 Introduction

The nuclear lamina was originally defined as a fibrous structure lining the inner nuclear membrane (INM), which is resistant to detergent and salt extraction [1]. This structure was subsequently shown to be composed of intermediate filaments (IF) which in amphibian oocytes formed into a basket weave network that interlinked nuclear pore complexes (NPCs) [2–4]. Now classified as type V IF proteins, the lamins are the evolutionary progenitors of the IF supergene family [5]. Lamins are divided into two sub-types, the A-type lamins and the B-type lamins. A-type lamins (lamins A, C, AΔ10 and C2) are alternatively spliced products of the *LMNA* gene [6]. During development they are generally expressed at the time of organogenesis [7], form relatively thick filaments [8] and are soluble during mitosis [9]. The B-type lamins (lamins B1, B2 and B3) are the products of two distinct genes. Lamin B1 (in humans) is encoded by the gene *LMNB1*, located on chromosome 5q23.3-31.1, whilst lamins B2 and B3 are alternatively spliced products of *LMNB2* located on chromosome 19p13.3 [10,11]. B-type lamins are anchored to the inner nuclear membrane *via* a prenylated cysteine residue at the C-terminus (see below) and are therefore associated with membranes during mitosis [9]. In addition, whilst lamin B3 is germ line specific, lamins B1 and B2 are expressed in most cells in embryos and adult animals [12–15], giving rise to the notion that they are essential for cell survival.

Like all IF proteins, lamins have a domain structure consisting of a (small) globular head domain, a central rod domain consisting of four coiled coil dimers and a (long) C-terminal globular domain. Lamins form obligate dimers (most likely homodimers), which then assemble into homopolymers. Lamin dimers are strongly predisposed towards proto-filament assembly *via* head-to-tail associations, which in turn form anti-parallel out of register lateral associations, eventually giving rise to 10–13 nm filaments [2,16,17].

The B-type lamins and prelamin A possess a CaaX motif that is a site for posttranslational modification. The CaaX motif is sequentially modified following translation by the addition of a 15-carbon farnesyl isoprenoid to the cysteine residue, proteolytic cleavage of the aaX residues and methylation of the now C-terminal cysteine. This renders the C-terminal domain hydrophobic and anchors permanently farnesylated lamins to the INM. Lamin A, however, undergoes further post-translational modification at the INM where the final 15 C-terminal amino acids are cleaved by the ZMPSTE24 endoproteinase, allowing the protein to migrate between the nucleoplasm and the lamina (see Levy and colleagues this edition, reviewed by Broers et al. [18]).

## 2 Lamina function in nuclear assembly, DNA replication, transcription and mitotic spindle assembly

The very first investigations indicating a functional role for B-type lamins arose from studies using cell-free extracts of *Xenopus* eggs that support the assembly of replication competent nuclei *in vitro* (Table 1). Physical or functional depletion of endogenous B-type lamins from these extracts led to the assembly of small fragile nuclei having functional nuclear pore complexes but which lacked a lamina and were unable to replicate DNA [19–21]. The mechanism by which lamins promote DNA replication in this system remains contentious and using similar approaches, some studies suggested that lamins assist in the elongation phase of DNA replication [22,23], whilst others have suggested that they contribute to the initiation phase of DNA replication [24,25]. Dominant negative lamin mutants can be used to disrupt pre-existing lamina filaments. When B-type lamina filaments are disrupted in this way in Xenopus oocytes, this leads to the sequestration of RNA polymerase II and inhibition of pol II dependent transcription [26]. B-type lamins have also been reported to interact with the NPC protein Nup153. Nup153 is a component of the nuclear pore basket which resides at the inner nuclear membrane. Depletion of either B-type lamins or Nup153 does not lead to gross abnormalities in NPC assembly or function but does lead to a phenotype in which NPCs float within the NE and cluster together [27]. Taken together these studies suggest that whilst B-type lamins are not essential for the assembly of transport competent NEs, they do have essential roles in NE integrity, maintaining the positioning of NPCs and in DNA replication and transcription. These essential functions of B-type lamins were supported by early siRNA depletion studies in somatic cells. These studies identified B-type lamins as essential for cell survival and their depletion led to apoptotic cell death [28].

More recently, B-type lamins have been implicated in the correct assembly and maintenance of mitotic spindles. Xenopus lamin Liii was detected as a matrix-like component of mitotic spindles in Xenopus egg extracts. Moreover, depletion of lamin B1 and lamin B2 from somatic cells led to a number of spindle defects including unfocused spindle poles, poor spindle morphology and lack of chromosome congression [29]. B-type lamin association with mitotic spindles is dependent upon RanGTP, Nudel and dynein and appears to occur at prometaphase [30,31]. B-type lamins appear to antagonise kinesin Eg5 to restrain spindle pole separation prior to anaphase and thus ensure correct spindle orientation and function [32] (Table 1).

## 3 Lamin B1 and cellular senescence

**Fig. 1:**
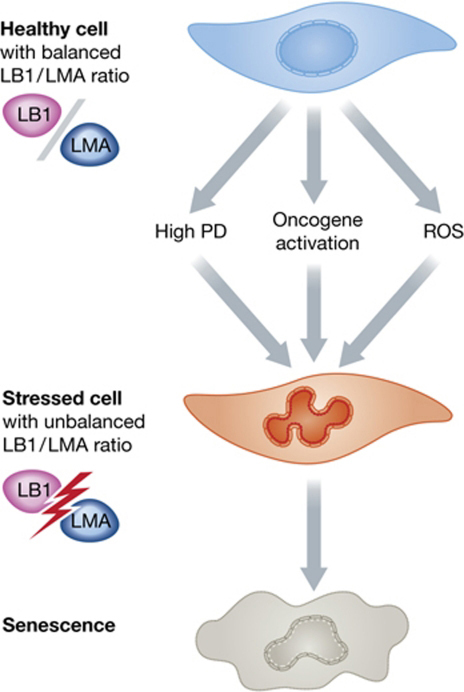
The effects of different cellular stresses that lead to senescence on lamin A to lamin B1 ratios. Healthy early passage cells have a balanced LMA to LB1 ratio. In response to senescence-inducing events, LB1 expression is altered and/or LMA is oxidized. This figure was reproduced with kind permission from EMBO Journal.

Recent reports have implied that changes in expression levels of lamin B1 are a hallmark of cellular senescence. Cellular senescence is a state of permanent cell cycle arrest triggered by a number of stresses including extended cell proliferation, persistent DNA damage, oxidative stress and oncogene activation. This in turn leads to activation of tumour suppressor genes, including p53 and Rb *via* p16 or p21 [33]. Recently it has been reported that lamin B1 accompanies replicative and oncogene induced senescence and that silencing of lamin B1 expression slowed proliferation rates and promoted cellular senescence *via* p53 and Rb dependent mechanisms [34]. In a complementary study Freund and co-workers also reported that loss of lamin B1 expression was a hallmark of senescence *in vitro* and *in vivo* but were equivocal as to whether this loss of expression was a cause or a consequence of senescence [35]. In an apparently contradictory study, entry into a senescent state as a result of MAPK activation and oxidative stress was accompanied by up-regulated expression of lamin B1 suggesting a novel mechanism of ATM-independent stress sensing [36]. The apparent discrepancies between the two studies was seemingly resolved by Dreesen and co-workers who showed that indeed up-regulation or down-regulation of lamin B1 expression could accompany cellular senescence but that lamin B1 silencing whilst causing cell cycle arrest did not in itself give rise to senescence. However, lamin B1 silencing in the presence of partial silencing of A-type lamins did [37]. This observation is apparently crucial, since replicative senescence is also accompanied by progressive oxidation of the lamin A tail leading to its functional inactivation [38]. Thus it appears that the ratio of lamin B1 to lamin A/C expression is critical and might be a key component of cellular ageing [39] (Fig. 1).

## 4 Lamin B1 and gene silencing

Lamin B1 has been implicated with gene silencing *via* a number of studies. It has been known for decades that heterochromatin preferentially associates with the nuclear lamina. More recently, it has been shown that lamin B1 associates with large lamina-associated domains (LADs) of chromatin which are devoid of active histone marks but enriched for repressive marks [40,41]. LADs are in general gene-poor, consistent with the idea that chromosomes which are preferentially associated with the nuclear periphery are also gene poor [42]. Forced repositioning of genes to the nuclear lamina, leads to transcriptional silencing [43] implying that the lamina is a silencing environment. Finally, whilst in general LADs maintain their lamina association during mouse ESC differentiation, local gene repositioning to the nuclear interior does occur and this is correlated with gene activation [44]. Taken together these observations suggest that the lamina is a repressive environment and have given rise to the notion that lamin B1 is involved in gene silencing. However, when ESCs devoid of B-type lamins (see section on mouse models) are induced to differentiate into trophectoderm altered patterns of gene expression and repositioning of genes occurs as efficiently as in wild type ESCs implying that B-type lamins do not directly regulate gene expression [45].

Redistribution of heterochromatin from the nuclear periphery to senescence-associated heterochromatic foci (SAHFs) is also a hallmark of cellular senescence. Since down-regulation of lamin B1 accompanies cellular senescence [34–37] it is possible that this contributes to SAHF formation. Two separate studies have revealed quite subtle changes in gene associations with the nuclear lamina during senescence, which depend upon the type of histone marks present. Genome wide mapping demonstrated that during senescence, lamin B1 is preferentially depleted from LADS enriched for H2K9me3 and that *LMNB1* silencing promotes the relocation of H2K9me3 from a predominantly perinuclear distribution to SAHFs [46]. However, despite a global reduction in lamin B1 levels in senescent cells lamin B1 binding to a sub-set of genes enriched for H3K27me3 increases and those genes become repressed [46,47]. Both studies suggest that lamin B1 depletion contributes to global chromatin reorganisation during senescence, although neither study demonstrates that lamin B1 contributes directly to gene silencing.

## 5 Studies in whole organisms

The notion that B-type lamins have essential functions and are therefore required for cell survival has been questioned by studies in whole organisms and their fundamental roles have now been studied in *Caenorhabditis elegans*, Drosophila and mouse models. Drosophila expresses two lamin sub-types, *LamDm0*, which is a ubiquitously expressed B-type lamin and *LamC* which is developmentally regulated [48]. Depletion of *LamDm0* using RNAi leads to a range of severe developmental phenotypes, although some individuals survive to late pupal stages or early adulthood [49]. One problem with studies in Drosophila is that eggs maintain a pool of maternal LamDm0 protein which could contribute to early development. In addition, expression of *LamC* during larval stages could compensate for *LamDm0* depletion. Nevertheless, it is clear that absence or depletion of *LamDm0* did not cause gross NE defects or loss of cell proliferation in a majority of tissues suggesting that these lamins had more subtle roles in tissue building rather than basic functions in DNA replication and transcription.

Similar results were obtained using RNAi mediated depletion of the single (B-type) lamin Ce-lam in *C. elegans*. Ce-lam depletion did lead to clear cell proliferation and chromosome segregation defects resembling a cut-phenotype in many cells. None-the-less whilst a majority of individuals were embryonic lethal, organogenesis did occur and a small number survived as short lived sterile adults [50,51]. Like Drosophila, *C. elegans* embryos possess maternally derived Ce-lam protein and this might account for the survival of some cells. However, it appears unlikely that the maternal pool could account for survival to adulthood, again suggesting that this B-type lamin is not required for all basic cellular functions but instead has roles in ensuring tissue building and integrity. Never-the-less, the prevalence of mitotic defects in embryos does imply that spindle assembly and function could be an important if not essential role for B-type lamins.

Studies in mice have reinforced the view that B-type lamins have important roles in building some if not all tissues and that these roles could be mediated *via* the interaction of these lamins with mitotic spindles. The earliest mouse model for lamin B1 function was an insertional mutant containing the first five *Lmnb1* exons joined in-frame to a *βgeo* reporter gene and thus lacked part of coil 2B and the entire C-terminal globular domain (termed *Lmnb1*^*Δ*^). Mice that were homozygous for this mutation were bred from heterozygotes and did not survive after birth. However, at embryonic day 18.5 they were present at an expected Mendelian ratio, indicating that mice could have been born live. *Lmnb1*^Δ/Δ^ mice were smaller than wild-types. Most internal organs were grossly normal but the lungs either lacked or had fewer alveoli, suggesting that death occurred through respiratory failure. Bone structure was grossly abnormal including curvature of the spine, decreased length of long bones and abnormal cranial structures. Mouse embryo fibroblasts (MEFs) derived from *Lmnb1*^Δ/Δ^ mice were able to proliferate and differentiate into adipocytes but became polyploidy and senesced prematurely. Taken together these results indicate that lamin B1 deficiency does not affect cell survival and differentiation *per se* but does affect the development of certain tissues and has important functions in maintaining genome stability in somatic cells [52].

More recently a lamin B2 deficient mouse model has been produced by inserting a *LacZ* reporter into exon 1 of *Lmnb2*. This mutant specifically disrupts lamin B2, since the sperm specific lamin B3 uses an alternative exon1. *Lmnb2*^*−/−*^ mice were bred from *Lmnb2*^*+/−*^ mice and yielded homozygotes at expected Mendelian ratios. *Lmnb2*^*−/−*^ mice were born but died within one hour. The mice had normal body weights and did not display any abnormalities in organ systems except the brain, which displayed grossly abnormal neuronal layering in the forebrain. More detailed investigations revealed that at E13.5 the neocortex appeared normal but subsequent neuronal migration into the cortex was impaired. MEFs derived from *Lmnb2*^*−/−*^ mice displayed no growth defects, suggesting a highly specific role for lamin B2 in neuronal migration [53]. A more recent study investigated the role of farnesylation of B-type lamins in neuronal migration. Knock-in mice expressing non-farnesylated versions of lamin B1 or lamin B2 were created. In contrast, to *Lmnb2*^*−/−*^ mice, mice expressing non-farnesylated lamin B2 developed normally and were apparently healthy. Mice expressing non-farnesylated lamin B1, however, died at birth and had striking neuronal defects. In particular, in migrating neurones the nuclear lamina was apparently torn away from chromatin, which was left ‘naked’ [54]. Thus, lamin B1 also appears to have a role in neuronal migration.

Whilst these mouse models suggest specific and overlapping functions for each B-type lamin, it has been argued that in the case of single knock-out mice the remaining B-type lamin could compensate for the lack of the other and therefore mask additional fundamental roles. To investigate this hypothesis, Embryonic Stem Cells (ESCs) that were null for lamin B1 and B2 were created from blastocysts obtained by mating *Lmnb1*^+/−^ and *Lmnb2*^*+/−*^ mice. Since ESCs do not express A-type lamins, these cells were effectively null for all lamin sub-types. Surprisingly, these ESCs formed colonies and displayed growth rates that were similar to wild type ESCs, expressed pluripotency markers and maintained ploidy, suggesting that lamins are not essential for ESC growth and survival. The *Lmnb1*^*−/−*^*Lmnb2*^*−/−*^ ESCs were also able to differentiate into trophectoderm with similar gene expression changes to wild type ESCs. By crossing *Lmnb1*^*+/−*^*Lmnb2*^*+/−*^ mice, the expected Mendelian distribution of *Lmnb1*^*−/−*^*Lmnb2*^*−/−*^ embryos were observed from E12.5 to E18.5 but pups died immediately after birth through a failure to breath. The mice displayed multiple organ defects including poorly developed lungs, diaphragms and brains. However, other organs systems such as heart were normal. Similar to the *Lmnb2*^*−/−*^ mice, brain abnormality in the double knock-out mice appeared to occur because of grossly impaired neuronal migration. The ability of some organ systems to develop normally in this mouse model might be explained because A-type lamins are more critical for the maintenance of some organs. It is intriguing to note from this perspective that in the lung and in neurones, A-type lamins are absent or expressed at very low levels [45].

The finding that B-type lamins are essential for the development and maintenance of some tissues but not others was reinforced by the finding that a conditional double knockout of *Lmnb1* and *Lmnb2* in keratinocytes has no deleterious effects. The mice developed normally and had normal lifespans with no gross abnormalities in skin morphology, hair production or production of nails throughout life. Moreover, unlike *Lmnb1*^*Δ/Δ*^ MEFs, *Lmnb1*^*−/−*^*Lmnb2*^*−/−*^ keratinocytes grew normally, had some abnormalities in nuclear morphology but did not display aneuploidy [55]. Similar results were obtained in a cre-recombinase knockout of *Lmnb1* and *Lmnb2* in hepatocytes. Hep-*Lmnb1*^*−/−*^*Lmnb2*^*−/−*^ mice had normal liver function and morphology and in tissues had no nuclear defects. Isolated hepatocytes did display nuclear shape abnormalities but this did not reflect the *in vivo* situation [56]. Thus B-type lamins appear to be completely dispensable in some quite complex organ systems (Table 2).

## 6 The role of B-type lamins in human disease

It is now well established that mutations in *LMNA* give rise to a greater variety of genetic disorders than any other known gene (reviewed by Broers et al. [18], and other reviews of this edition: Azibani et al., Guénantin et al., Cau & Levy). In contrast, mutations in *LMNB2* have yet to be directly associated with any human disease (although *LMNB2* variants have been implicated in acquired partial lipodystrophies [57,58] see Guénantin et al., this edition), whilst diseases associated with *LMNB1* are just emerging. Adult-onset autosomal dominant leukodystrophy (ADLD) is a very rare demyelinating neuropathy of the central nervous system that presents in the fourth or fifth decade of life. The disease is usually but not always associated with early autonomic symptoms and eventually ataxia [59–61]. Cardiovascular and skin defects have also been reported in one ADLD family leaving the possibility of additional hallmarks of the disease [62]. In ADLD patients, white matter defects are observed, particularly in the cerebellum, corticospinal tracts and corpus callosum, which leads to brain and spinal cord atrophy [63]. At a histological level, these lesions display astrogliosis but oligodendrocyte preservation [64].

ADLD has been shown to be caused by duplications involving *LMNB1* on chr. 5q32 [59]. Duplication boundaries have now been mapped in a large collection of ADLD families, confirming that the minimal duplicated regions necessary for the disease includes the whole of the *LMNB1* gene, which can be inverted, and that probably arose through non-homologous end joining or replication-based defects [65]. Duplication of *LMNB1* leads to increased lamin B1 mRNA and protein expression in brain tissue. It has been proposed that somehow, increased expression of lamin B1 causes suppressed transcription and lack of myelin basic protein and proteolipid protein in oligodendrocytes, inducing central myelin breakdown. *LMNB1* is negatively regulated by miR-23, which is also implicated in ADLD variants and it has been proposed that an over representation of lamin B1 mRNA sequesters miR-23 leading to disturbances in myelin protein production [60,66].

More recently, *LMNB1* has been implicated as a susceptibility gene in neural tube defects. In a mouse model of spina bifida and exencephaly (*curly tail*), a *Lmnb1* polymorphic variant was implicated as a modifier of neural tube closure defects. The defect results in loss of a C-terminal glutamic acid residue that causes nuclear shape defects and premature senescence in MEFs. Crossing *curly tail* strains with wild-type *Lmnb1* strains partially rescues spina bifida and exencephaly [67]. In a study involving 239 patients with ADLD, exon sequences revealed a number of polymorphisms in *LMNB1*, including five synonymous and three non-synonymous (missense) variants. Of these, p.A501V was detected in nine patients, whilst p.A436T and p.D448G were each only detected in one patient. Expression of p.A436T in HeLa cells promoted nuclear dysmorphism [68]. Whilst not definitive, these intriguing results indicate that *LMNB1* might contribute to neural tube defects in humans.

More recently, *LMNB1* has also been implicated in cancers. In combination with vimentin, circulating lamin B1 is diagnostic of early stage hepatocellular carcinomas [69,70]. In breast cancer, decreased expression of *LMNB1* is associated with poor prognosis [71]. This study is consistent with a finding that over-expression of lamin B1 is associated with low grade differentiation in pancreatic cancer and that the drug betulinic acid down-regulates lamin B1 expression as part of its anti-cancer activity [72]. Finally, β-Asarone induces senescence in colorectal cancer cells by inducing lamin B1 over-expression [73]. Taken together, these studies suggest that lamin B1 might be an important new target for anti-cancer therapies in a range of epithelial tumours.

## 7 Conclusions

For years it was thought that B-type lamins had essential functions in processes associated with cell proliferation. The original studies supporting these views were carried out in highly specialised model systems with either extremely simplified nuclear envelopes (Xenopus eggs) or highly specialised chromosome organisation (Xenopus oocytes). These systems also express a single specialised germ line specific B-type lamin (lamin Liii – [19,20]). In contrast, complete elimination of B-type lamins from mouse ESCs has apparently few deleterious effects. Their elimination from developing embryos certainly causes lethality, but some tissues, particularly brain, lung and bones, appear more vulnerable to such loss than others (*e.g.* skin and liver) [33,71,56]. Altered lamin B1 expression is also implicated in cellular senescence where it might contribute to the formation of SAHFs [46]. However, in both mouse development and senescence it appears that any deleterious function resulting from reduced lamin B1 expression is also associated with reduced or absent expression of lamin A/C implying that serious problems only arise when A-type lamins cannot compensate for loss of B-type lamin expression [37,45]. This is most dramatically seen in the brain where loss of lamin B2 expression or altered lamin B1 farnesylation causes severe loss of brain organisation and neuronal migration in mice [53,54], whilst *LMNB1* duplication leads to severe adult onset brain and CNS degeneration in humans [59]. The molecular mechanisms behind these effects remain unclear but appear to arise through direct effects on neuronal nuclear structure [54] or indirect effects on oligodendrocyte mediated neuronal myelination [60]. Future studies should focus on lamin B1 interactions (*e.g.* with miR-23) which will form the basis of understand the pathways that do depend on these lamins.

## Figures and Tables

**Table 1:** The effects of lamin depletion or knock-down, on sub-cellular organisation or cellular function.

Experimental system	Lamin sub-type	Cellular defects	Reference
Xenopus egg extracts	Lamin Liii	Fragile nuclei, DNA replication failure, NPC positioning, Spindle assembly defects	[19,20,27,29]
Xenopus oocytes	Lamin Liii	Pol II transcription failure	[26]
HeLa cells	Lamin B1 and Lamin B2	Apoptosis	[28]

**Table 2:** The effects of depletion or knock-out of [74] lamin sub-types on cellular function and organogenesis in *C. elegans*, *Drosophila melanogaster* and mouse.

Organism	Lamin sub-type	Cellular defects	Organ defects	Reference
*C. elegans*	Ce-lam	Chromosome segregation	Multiple embryonic defects, adult sterility	[74,51]
*Drosophila*	LamDm0	None	Multiple	[49]
Mouse	Lamin B1	Polyploidy, premature senescence	Small size, lung and bone, particularly spine and cranium	[52]
Mouse	Lamin B2	Nuclear shearing in migrating neurones	Neuronal layering in forebrain	[53]
Mouse	Lamin B1 and B2	None is ESCs	Small size, lung, bone and brain development	[45]
